# Analysis of socioeconomic differences in the quality of antenatal services in low and middle-income countries (LMICs)

**DOI:** 10.1371/journal.pone.0192513

**Published:** 2018-02-23

**Authors:** Joshua Amo-Adjei, Kofi Aduo-Adjei, Christiana Opoku-Nyamah, Chimaroake Izugbara

**Affiliations:** 1 African Population and Health Research Centre, Nairobi, Kenya; 2 Department of Population and Health, University of Cape Coast, Cape Coast, Ghana; 3 Institute of Demography, National Research University Higher School of Economics, Moscow, Russia; 4 Department of Geography and Regional Planning, University of Cape Coast, Cape Coast, Ghana; Rudjer Boskovic Institute, CROATIA

## Abstract

The desired results of increasing access and availability of antenatal care (ANC) services may not be realized if the quality of care offered is not adequate. We analyzed the content/quality of antenatal care to determine whether there are socioeconomic (education and wealth) inequalities in the services provided in 59 low and middle income countries in six WHO regions–Africa, East Asia and Pacific, Europe and Central Asia, Latin America and Caribbean, Middle East and South Asia. We aggregated the most recent (2005–2015) Demographic and Health Survey for each country. The quality of content was measured on eight recommended ANC services–(1) monitoring of blood pressure; (2) tetanus injection; (3) urine analysis for protein; (4) blood test; (5) information about danger signs (6); weight (7); height measurements and (8) provision of iron-folate supplement. Descriptive and Poisson regression techniques were applied to analyse the data. We found considerable wealth and educational differences prior to controlling for known covariates. Between wealth and education, however, the disparities in the latter are larger than the former. Whereas the socioeconomic differences remained at post adjusting for residence, place and number of antenatal care, parity and region, the magnitude of change was minimal. Higher number of ANC content was provided in “other” forms of private facilities; the Latin America and Caribbean region recorded the highest number of content compared to the other regions. The hypothesized socioeconomic status on content/number of ANC services was generally supported, although the associations are substantially constrained to other variables. Efforts are made to increase the number and timing of ANC services; due recognition is needed for the content offered.

## Introduction

The recently launched Sustainable Development Goals (SDGs) affirm the very urgent need to tackle the root causes of maternal and child morbidity and mortality. Apart from being a desirable goal in its right, improved maternal health is also a fulcrum for healthier child development, holding the key to equity, lifelong health, wellbeing, and productivity. [1]. Specifically, SDG target 3.8 aims at “achieving universal health coverage, including financial risk protection, access to quality essential health-care services and access to safe, effective, quality, and affordable essential medicines and vaccines for all” [2]

An avalanche of research evidence support the notion that achieving quality maternal and child health outcomes is firmly hinged on prenatal health conditions of mothers [3,4]. One of the proven interventions to identify and guarantee the health of mothers and eventually, their children during and after the pregnancy period is antenatal care (ANC). ANC has noted several benefits, including but not limited to, preparation for birth, prevention and recognition and controlling of pregnancy complications, early detection of pre-existing conditions that can deteriorate during pregnancy (e.g. diabetes and hypertension), poor lifestyles [5,6] and skilled birth attendant [7], which are crucial to averting and or managing intrapartum complications.

Quite a significant progress has been made in expanding and availing maternity services–pre, intra- and postpartum to women in developing and lower-middle income countries. Concerning pre-partum or antenatal care for instance, about 52% of women in 2014, compared to 35% in 1990 within developing countries received adequate–indicated as ANC visits of four or more during pregnancy [8], making the full realization of ANC benefits an “unfinished business”

While efforts are underway to universalize improvements in ANC coverage and utilization, research evidence suggests significant gaps in contact and exploitation of antenatal health services due to individual women’s socioeconomic status (SES) and are exacerbated by structural weaknesses (e.g. poor infrastructure, shortage of qualified health personal as well as unavailability of functional equipment for delivery of services) [9,10]. As efforts are underway to encourage and increase access to early ANC initiation and for women to achieve a minimum of eight visits, it is appropriate to assess whether the services women obtain measure up to the recommended content of care, an indication of quality.

The *2000* World Health Report devoted substantial portions to making health systems respond to users’ expectations [11]. Following the report, studies on health systems responsiveness, in part, viewed as the quality of care has increased in the last two decades [12,13]. Quality of health care is generally framed as the level of service provision that meets the expectations of individuals and patients [14]. According to Donabedian [15], quality of care must have the following: (i) *structure* (facility infrastructure, management and staffing), (ii) process (technical/functional quality and patient experience) and (iii) outcomes (patient satisfaction, return visits and health outcomes). The existing literature reveals that quality of care studies tend to focus on outcomes (usually relational care), drawing largely on user notions of quality [16,17].

We analysed the quality of ANC provided in public, private and faith-based health facilities in low and middle income countries (LMICs) in Africa, Asia, Europe, Latin America and the Caribbean and the extent to which women’s socioeconomic status (wealth and education) contributed to the observed situation. To our knowledge, no previous study has utilized comparable nationally representative datasets to analyse SES inequalities in the functional/technical quality of ANC services in LMICs. This is important for tracking and understanding progress towards improved better maternal and child health outcomes, particularly in the light of the global community’s ambitious agenda of “leaving no one behind” [18].

## Data and methods

### Data

We extracted the most recent nation-wide household demographic and health survey (DHS) data from 59 countries in Africa, East Asia and Pacific, Europe and Central Asia, Latin America and Caribbean, Middle East and South Asia, categorizes based on WHO regions. Our analysis was restricted to live births that occurred either in the three or five years to the survey. From the 5^th^ round of the surveys, the DHS programme extended the collection of records on births to those occurring in five, instead of three years. We also limited the analysis of ANC records to the most recent births among women who reported more than one birth in the either three or five year period before the survey. The DHS uses similar sampling processes and interview modules, making cross-country comparability feasible. The methods are documented in previous studies [19,20].

### Study variables

Our dependent variable was the quality of ANC derived from the WHO clinical guidelines for focused ANC, spread across a minimum of four visits [21], which has recently been revised upwards to eight by the WHO [22]. The quality of content was measured on eight WHO recommended ANC service elements–(1) monitoring of blood pressure, (2) tetanus injection, (3) urine analysis for protein, (4) blood test, (5) information about danger signs (6), weight and (7) height measurements and (8) provision of iron-folate supplement. From these eight variables, we created a count score, ranging from 0–8 with eight indicating that the woman received care on all the indicators. Intermittent preventive therapy for malaria was excluded from the analysis since it is not endemic in all the 59 countries considered for our analysis.

Following some previous studies [23,24–26], we used household quintile (poorest, poorer, average, richer and richest) and maternal education (no education, primary, secondary and higher) as measures of SES. Apart from these factors, we controlled for residence (urban-rural), parity (nulliparous, multiparous and grandparous), place of ANC–dummy for home, government hospital, government center, maternity clinic, village health unit, other public facility, other private facility, private health center, and religious hospital, timing of first ANC (first trimester; after first trimester) and number of ANC visits (less than 4 and 4 or more), region–grouped based on WHO categorisation. These are: Africa, East Asia and Pacific, Europe and Central Asia, Latin America and Caribbean, Middle East and South Asia.

### Analytical strategy

We utilised graphs and Poisson regression to present the data. Specifically, we show mean number of ANC services women received by regions, educational attainment of women as well as their wealth status. We then applied a Poisson regression because the main outcome variable was constructed as count. The coefficients were then Exponentiated into odds ratios since the coefficients say very little in respect of explanation. To adequately explicate the SES inequalities in the quality of service provided, we estimated two models with the first one involving only SES variables while a second was modelled to include the control variables. Next, we computed marginal effects of wealth and education alone and a second set of marginal effects with wealth, education and all the control variables. We applied individual weighting factors to the analysis. The weighting factors are derived from the household weight multiplied by the inverse of the individual response rate of the individual response rate group [27].

## Results

**Fig 1:**
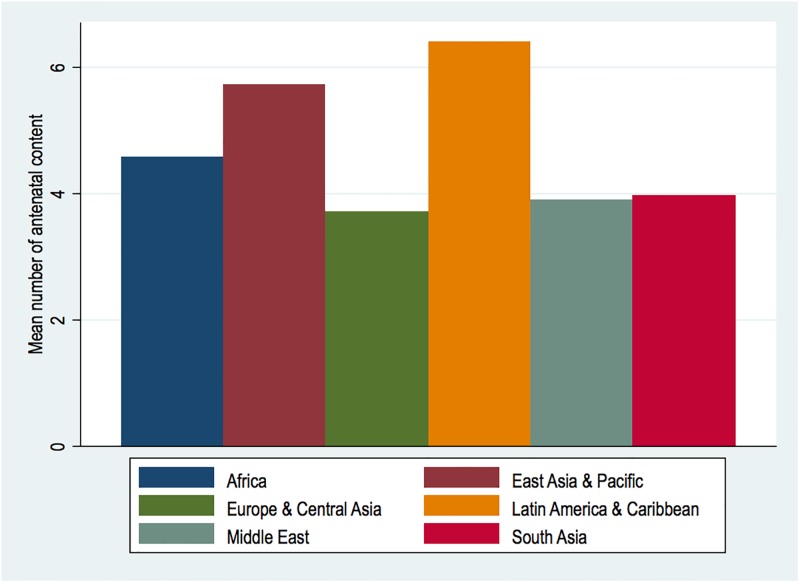
Mean number of antenatal care services by regions.

There were 400,336 weighted women with recent records on births, drawn from 32 countries in Africa (N = 224,772; 56.15%), six in Europe and Central Asia (N = 11,385; 2.84%), eight Latin America and Caribbean countries (N = 45,837; 11.45%) and four each from East Asia and Pacific (N = 31.793; 7.94), Middle East (N = 35,628; 8.9%) and South Asia (N = 50,918; 12.72%). In all, women received around 4.71 (4.70–4.72) services averagely, with regional range from 3.71–6.39 in Europe and Central Asia and Latin America and Caribbean, illustrated in Fig 1.

**Fig 2:**
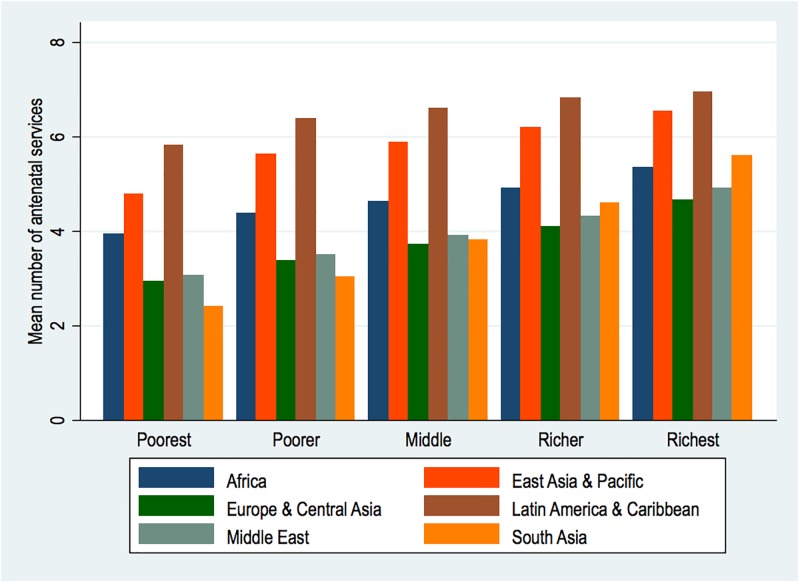
Mean number of antenatal care services by wealth and regions.

Fig 2, shows the average number of services by wealth in each of the regions. It is evident that at every level of wealth, Latin America and Caribbean women fared better or obtained more services than women in the other regions although the differences do not graduate substantially with rising wealth. However, the sharpest poor-rich gaps are noted in South Asia, where the poorest average approximately two contents, with the richest averaging five services.

**Fig 3:**
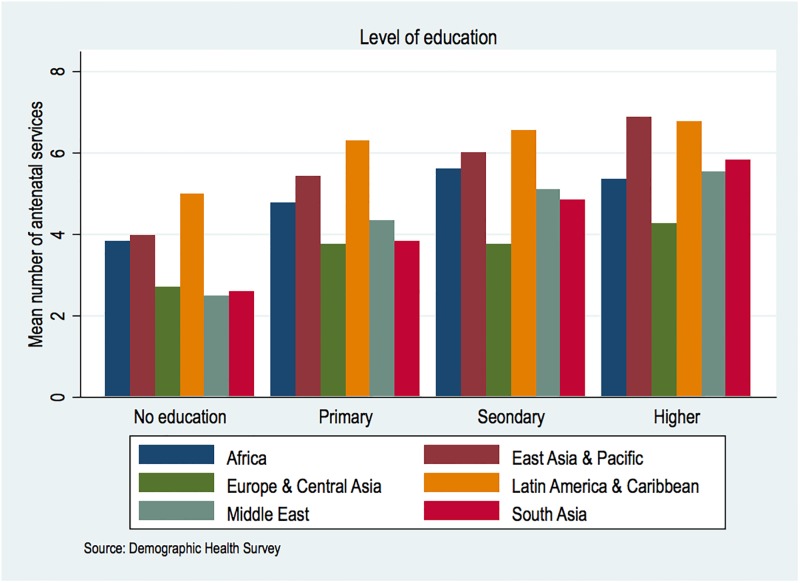
Mean number of antenatal services by education in the six regions.

Illustrated in Fig 3 are average ANC contents reported by women across different levels of education over six regions. The overall number rises with education but some areas are relatively outstanding than others. For instance, women with no formal education recorded more services than women with higher education in Europe and Central Asia regions. Noteworthy also is the marginal variations in the number of ANC services by education among European and Central Asian countries studied, which ranged from 2–4.2. Responding women with higher education in East Asia and Pacific had the highest number of services.

### Regression analysis

#### Regional variations

In the models involving education and wealth alone, the Exponentiated coefficients varied across regions. For the highest wealth quintile, the probability of women receiving more services ranged from 1.26–1.76 compared to the poorest in Africa and South Asia. Adjusted for other determinants, the odds varied between 0.96–1.52, gauged against the poorest in Africa and South America & Caribbean. Regarding education, the odds of women receiving more content between the highest educated women and those reporting no education (reference group) bounded within 1.25–2.01 in Latin America, and Caribbean and Middle East. Remodeling with the other covariates, the disparities between lowest-highest educational levels ranged from 1.01–1.58 in East Asia and Pacific on the one hand and Europe & Central Asia on the other (Data not shown).

#### Combined findings

**Fig 4:**
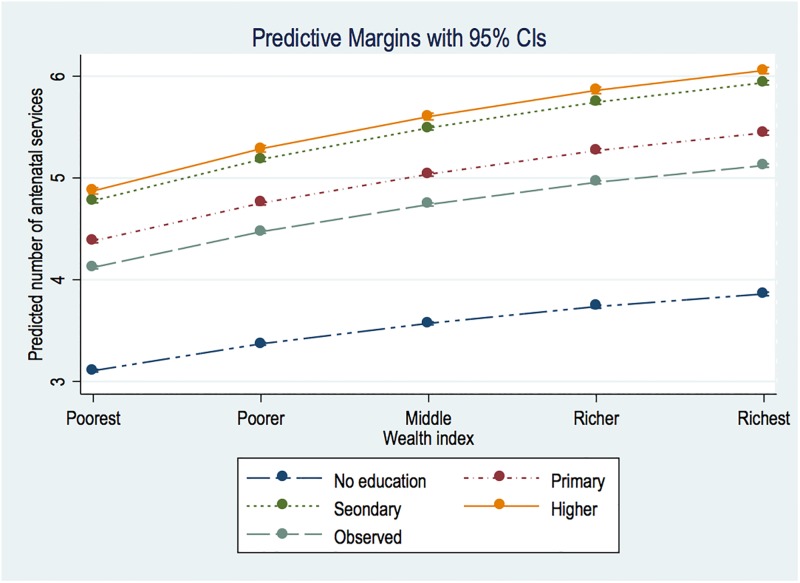
Education and wealth margins plot.

The pooled results for the 59 countries are displayed in Table 1. As shown in Model 1, the richest group, compared with the poorest, received 24% more services than the poorest. The differences in education, no *vs*. higher are also significant–with the latter receiving roughly 57% more than the former. In Fig 4, we clarify the wealth and education matrix through marginsplot, which shows a consistent linear SES slope. However, the interval between secondary and higher educated women is significantly close.

**Table 1:** Poisson regression results on the number of ANC services provided in 59 LMICs in periods 2005–2006 and 2014–2015.

	Model 1		Model 2	
	IRR	95% CI	IRR	95% CI
Content				
**Wealth Quintile** (Poorest)	1	[1,1]	1	[1,1]
Poorer	1.085***	[1.080,1.090]	1.015***	[1.010,1.020]
Average	1.150***	[1.144,1.155]	1.023***	[1.018,1.028]
Richer	1.203***	[1.197,1.208]	1.029***	[1.024,1.035]
Richest	1.243***	[1.237,1.249]	1.024***	[1.018,1.030]
**Education** (None)	1	[1,1]	1	[1,1]
Primary	1.410***	[1.405,1.416]	1.088***	[1.083,1.092]
Secondary	1.538***	[1.532,1.544]	1.123***	[1.118,1.128]
Higher	1.569***	[1.559,1.578]	1.094***	[1.087,1.101]
**Residence** (Urban)			1	[1,1]
Rural			0.938***	[0.934,0.941]
**Home ANC** (Yes)			0.919***	[0.908,0.930]
**Government hospital** (Yes)			1.283***	[1.277,1.288]
**Government Health Center** (Yes)			1.268***	[1.262,1.274]
**Maternity Clinic** (Yes)			1.176***	[1.169,1.182]
**Village Health Unit** (Yes)			1.137***	[1.128,1.146]
**Other Public** (Yes)			1.125***	[1.116,1.133]
**Other Private** (Yes)			1.388***	[1.373,1.404]
**Private Health Clinic** (Yes)			1.245***	[1.238,1.252]
**Religious Hospital** (Yes)			1.316***	[1.304,1.327]
**Timing of first ANC** (First trimester)			1	[1,1]
Second trimester ANC			0.931***	[0.927,0.934]
**Number of ANC** (≤3)			1	[1,1]
≥4			1.161***	[1.157,1.166]
**Parity** (Nulliparous)			1	[1,1]
2–5			0.999	[0.996,1.003]
≥6			0.984***	[0.980,0.988]
**Region (Africa)**			1	[1,1]
East Asia and Pacific			1.130***	[1.124,1.136]
Europe and Central Asia			0.770***	[0.764,0.776]
Latin America and Caribbean			1.149***	[1.144,1.155]
Middle East			0.827***	[0.822,0.833]
South Asia			0.887***	[0.882,0.892]
Constant	3.106***	[3.094,3.119]	3.857***	[3.826,3.888]
Log lik.	-953448.7		-733234.8	
Chi-squared	86126.7		81211.2	
*N*	403,258		354,136	

Exponentiated coefficients; 95% confidence intervals in brackets

* *p* < 0.05,

** *p* < 0.01,

*** *p* < 0.001

**Fig 5:**
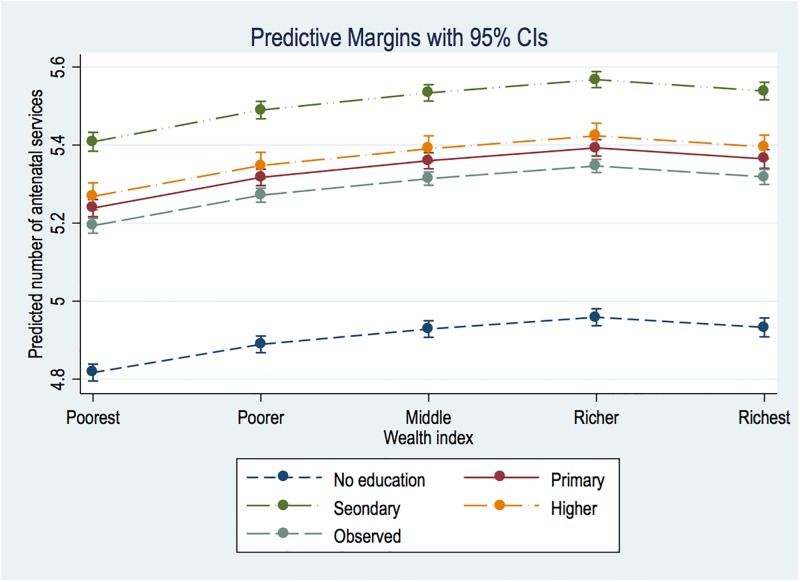
Education and wealth margins plot from multivariate analysis.

In the adjusted Model (2), the magnitude of SES differences are almost wiped out, with richer women having only 2.9% advantage and the most educated having a 9.4% edge over those with no formal education. Contrary to observations in Fig 4, that of Fig 5 reveals relatively wide margins in favour of secondary than higher educated women.

Other than the SES constructs, ANC contacts in “Other Private”, Religious and Private Health Clinics increased content by 39%, 32% and 24.5%, respectively. Appraised by geographic regions, East Asia/Pacific and Latin America and Caribbean women reported 13% and 15% more services but studied countries from Europe/Central Asia, Middle East and South Asia were materially less likely to receive higher number of services compared to women in Africa.

## Discussion

Drawing on rich datasets that reflected nationally, we found that nearly 27% of critical ANC services were not provided to women in the countries studied. Also, in no region and country did women report obtaining all the eight recommended services. From the pooled multivariate estimates, SES differences are noted in content provided. However, in the Latin America and Caribbean region, the rich-poor gaps were not considerably substantial. In the remaining regions, it was very substantial, in excess of over 80% in some instances.

Earlier studies on inequities in ANC contacts highlight the peculiar disadvantages of the population lowest on SES measures. [28–31] While these SES differentials may possibly not arise from a systemic exclusion of the poor [10], they speak substantially about the fact that the poor experience a “double jeopardy”–limited coverage as well as poor quality services. In some respect, this may be explained as result of the number of ANC contacts, which indeed our results confirm. However, the types of services we analysed are so critical that efforts are made to ensure that content is improved for women, taking into consideration their peculiar dispositions. Positive interventions such as removal of maternity care costs are being pursued in some developing countries [32–35], so it is imperative to implement mechanisms that optimise ANC benefits to those at greater risk of missing out on the required number of contacts. By this, quality of care, rather than contacts may serve the needs of such women whose situation is not necessarily due to obvious avoidance of attending ANC but are pressed by both macro and micro systems/environments. Optimising the first ANC contact by cultivating the minds of pregnant women to the appropriate wide range of services at each visit will also be helpful.

The results showed significant gaps in the functional quality by facility ownership/management. Generally, leaders of private and religious facilities obtained more functional content than other providers. These differences may be linked to the heterogeneity of contexts, which drives the quality offered in public, private and religious and other facility categories. For instance, Victora et al. [10] found that the private sector offered higher functional quality than the public sector. Similar results have been reported in Gambia [36], Kenya [37] and Tanzania [38]. Contrary to these, few others have reported better quality performance in the public sector [39].

We noted strong association between early ANC and quality of care provided during the index pregnancy. We also found significant differences in parity–multiparous and grandparous women received lower quality than nulliparous women. The inter-linkages between these two variables in respect of ANC visits are well established, with evidence documenting how parity moderates early ANC initiation. Evidence also shows that late pregnancy recognition [40], low risk appreciation due to previous experiences [41] in addition to supply-side obstacles [42] contribute to this problem.

Another finding worth pointing out are the regional differences noted. The regional differences may be explained by the relative differences in the level of overall health services development and particular commitments to maternity care in general. For instance, some of the Middle East, South Asia and Central Asian countries in the sample have experienced substantial national political disruptions in the last decade. Evidence abounds about the wreckages that political instabilities cause in health systems, leading to poor maternal and child health indicators. On the other hand, the significance of higher quality in Latin America and Caribbean may be linked to the remarkable improvements in overall antenatal coverage since 1990. The region experienced large number of women receiving a minimum of four visits as of 2015 –from 75% in 1990 [43].

Despite the important findings presented here, some limitations of the study require mentioning. First, the range of services we analysed is not exhaustive. We focused on the eight services due, primarily to, data availability. However, we believe that the services–monitoring of blood pressure, tetanus injection, urine analysis for protein, blood test, information about danger signs and provision of the iron-folate supplement are of great importance in ANC service delivery. Second, the wide dissimilarities in national contexts of the countries rendered impossible inclusion of sociocultural variables such as religion and ethnicity. Nonetheless, by applying very familiar makers of ANC coverage and utilization, we are able to point certain variables that are generally amenable to broad policy interventions.

## Conclusion

We assessed some core elements of functional/technical quality of ANC services women reported in 59 LMICs. While most countries in these regions are making tremendous efforts to increase ANC coverage by reducing structural barriers to optimum utilization, it is imperative that the quality of care provided is closely monitored in ways that do not neglect women at the lower rungs of SES. Conscious steps are needed to reach out to women who, most likely, will still not meet the required number of contacts. In fact, evidence [44–46] from advanced countries where structural issues of accessibility barely exist, the complexities of cultural diversities hinder maximum utilization (early and number). The implication is that quality should not emanate solely from frequency of contacts. Improving functional quality is imperative for better maternal health outcomes.

## References

[1] Lawn JE, Blencowe H, Oza S, You D, Lee AC, Waiswa P, et al (2014) Every Newborn: progress, priorities, and potential beyond survival. The Lancet 384: 189–205. 10.1016/S0140-6736(14)60496-7 24853593

[2] UN Economic and Social Council (2016) Report of the inter-agency and expert group on sustainable development goal indicators.

[3] Gomez GB, Kamb ML, Newman LM, Mark J, Broutet N, Hawkes SJ (2013) Untreated maternal syphilis and adverse outcomes of pregnancy: a systematic review and meta-analysis. Bulletin of the World Health Organization 91: 217–226. doi: 10.2471/BLT.12.107623 23476094 10.2471/BLT.12.107623 PMC3590617

[4] Lindberg L, Maddow-Zimet I, Kost K, Lincoln A (2015) Pregnancy intentions and maternal and child health: an analysis of longitudinal data in Oklahoma. Maternal and child health journal 19: 1087–1096. doi: 10.1007/s10995-014-1609-6 25287250 10.1007/s10995-014-1609-6 PMC4388754

[5] Hughes RC, Moore MP, Gullam JE, Mohamed K, Rowan J (2014) An early pregnancy HbA1c≥ 5.9%(41 mmol/mol) is optimal for detecting diabetes and identifies women at increased risk of adverse pregnancy outcomes. Diabetes Care 37: 2953–2959. doi: 10.2337/dc14-1312 25190675 10.2337/dc14-1312

[6] Soma-Pillay P, Macdonald P. Early detection of Pre-Eclampsia: review; 2016 In House Publications pp. 5–8.

[7] Adjiwanou V, LeGrand T (2013) Does antenatal care matter in the use of skilled birth attendance in rural Africa: a multi-country analysis. Social science & medicine 86: 26–34. 23608091 10.1016/j.socscimed.2013.02.047

[8] Way C (2015) The Millennium Development Goals Report 2015: UN.

[9] Andrade MV, Noronha K, Singh A, Rodrigues CG, Padmadas SS (2012) Antenatal care use in Brazil and India: scale, outreach and socioeconomic inequality. Health & place 18: 942–950. 22832334 10.1016/j.healthplace.2012.06.014

[10] Victora CG, Matijasevich A, Silveira M, Santos I, Barros A, Barros F (2010) Socio-economic and ethnic group inequities in antenatal care quality in the public and private sector in Brazil. Health policy and planning 25: 253–261. doi: 10.1093/heapol/czp065 20123940 10.1093/heapol/czp065 PMC2889278

[11] World Health Organization (2000) The world health report 2000: health systems: improving performance: World Health Organization.

[12] Owili PO, Muga MA, Mendez BR, Chen B (2017) Quality of maternity care and its determinants along the continuum in Kenya: A structural equation modeling analysis. PLOS ONE 12: e0177756 doi: 10.1371/journal.pone.0177756 28520771 10.1371/journal.pone.0177756 PMC5433759

[13] Downe S, Finlayson K, Tunçalp Ö, Gülmezoglu AM (2017) Factors that influence the provision of good‐quality routine antenatal services: a qualitative evidence synthesis of the views and experiences of maternity care providers. The Cochrane Library.

[14] Baker A (2001) Crossing the quality chasm: a new health system for the 21st century. BMJ: British Medical Journal 323: 1192.

[15] Donabedian A (1988) The quality of care: how can it be assessed? Jama 260: 1743–1748. 3045356 10.1001/jama.260.12.1743

[16] Izugbara CO, Wekesah F (2017) What does quality maternity care mean in a context of medical pluralism? Perspectives of women in Nigeria. Health Policy and Planning: czx131–czx131. 10.1093/heapol/czx131 PMC5886285 29036530

[17] Kimweri A, Hermosilla S, Larson E, Mbaruku G, Kruk ME (2016) Service quality influences delivery decisions: A qualitative study on maternity care in rural Tanzania. Journal of Reproductive Health and Medicine 2: S11–S15.

[18] UN General Assembly (2015) Transforming our world: The 2030 agenda for sustainable development. A/RES/70/1, 21 October.

[19] Amo-Adjei J, Tuoyire AD (2016) Effects of planned, mistimed and unwanted pregnancies on the use of prenatal health services in sub-Saharan Africa: a multicountry analysis of Demographic and Health Survey data. Tropical Medicine & International Health 21: 1552–1561. 27671922 10.1111/tmi.12788

[20] Burke M, Heft-Neal S, Bendavid E (2016) Sources of variation in under-5 mortality across sub-Saharan Africa: a spatial analysis. The Lancet Global Health 4: e936–e945. doi: 10.1016/S2214-109X(16)30212-1 27793587 10.1016/S2214-109X(16)30212-1

[21] Organization WH, UNICEF (2015) Pregnancy, childbirth, postpartum and newborn care: a guide for essential practice. 26561684

[22] World Health Organisation (2016) WHO recommendations on antenatal care for a positive pregnancy experience. Geneva: WHO. 28079998

[23] Ronsmans C, Holtz S, Stanton C (2006) Socioeconomic differentials in caesarean rates in developing countries: a retrospective analysis. The Lancet 368: 1516–1523. 10.1016/S0140-6736(06)69639-6 17071285

[24] Joshi C, Torvaldsen S, Hodgson R, Hayen A (2014) Factors associated with the use and quality of antenatal care in Nepal: a population-based study using the demographic and health survey data. BMC Pregnancy and Childbirth 14. 10.1186/1471-2393-14-94 PMC3943993 24589139

[25] Doku DT, Neupane S (2017) Survival analysis of the association between antenatal care attendance and neonatal mortality in 57 low-and middle-income countries. International journal of epidemiology 46: 1668–1677. 29040531 10.1093/ije/dyx125 PMC5837573

[26] Rani M, Bonu S, Harvey S (2008) Differentials in the quality of antenatal care in India. International Journal for Quality in Health Care 20: 62–71. doi: 10.1093/intqhc/mzm052 18024998 10.1093/intqhc/mzm052

[27] Rutstein SO, Rojas G (2006) Guide to DHS statistics. Calverton, MD: ORC Macro.

[28] Pathak PK, Singh A, Subramanian S (2010) Economic inequalities in maternal health care: prenatal care and skilled birth attendance in India, 1992–2006. PloS one 5: e13593 doi: 10.1371/journal.pone.0013593 21048964 10.1371/journal.pone.0013593 PMC2965095

[29] Houweling TA, Ronsmans C, Campbell OM, Kunst AE (2007) Huge poor-rich inequalities in maternity care: an international comparative study of maternity and child care in developing countries. Bulletin of the World Health Organization 85: 745–754. doi: 10.2471/BLT.06.038588 18038055 10.2471/BLT.06.038588 PMC2636501

[30] Chama-Chiliba CM, Koch SF (2015) Utilization of focused antenatal care in Zambia: examining individual- and community-level factors using a multilevel analysis. Health Policy Plan 30: 78–87. doi: 10.1093/heapol/czt099 24357197 10.1093/heapol/czt099

[31] Singh A, Padmadas SS, Mishra US, Pallikadavath S, Johnson FA, Matthews Z (2012) Socio-economic inequalities in the use of postnatal care in India. PloS one 7: e37037 doi: 10.1371/journal.pone.0037037 22623976 10.1371/journal.pone.0037037 PMC3356397

[32] Witter S, Arhinful DK, Kusi A, Zakariah-Akoto S (2007) The experience of Ghana in implementing a user fee exemption policy to provide free delivery care. Reproductive Health Matters 15: 61–71. doi: 10.1016/S0968-8080(07)30325-X 17938071 10.1016/S0968-8080(07)30325-X

[33] Witter S, Adjei S, Armar-Klemesu M, Graham W (2009) Providing free maternal health care: ten lessons from an evaluation of the national delivery exemption policy in Ghana. Global Health Action 2. 10.3402/gha.v2i0.1881 PMC2779941 20027275

[34] Bellows NM, Bellows BW, Warren C (2011) Systematic Review: The use of vouchers for reproductive health services in developing countries: systematic review. Tropical Medicine & International Health 16: 84–96. 21044235 10.1111/j.1365-3156.2010.02667.x

[35] Ahmed S, Khan MM (2011) Is demand-side financing equity enhancing? Lessons from a maternal health voucher scheme in Bangladesh. Social Science & Medicine 72: 1704–1710. 21546145 10.1016/j.socscimed.2011.03.031

[36] Jallow IK, Chou Y-J, Liu T-L, Huang N (2012) Women’s perception of antenatal care services in public and private clinics in the Gambia. International Journal for Quality in Health Care 24: 595–600. doi: 10.1093/intqhc/mzs033 22789667 10.1093/intqhc/mzs033

[37] Agha S, Do M (2009) The quality of family planning services and client satisfaction in the public and private sectors in Kenya. International Journal for Quality in Health Care 21: 87–96. doi: 10.1093/intqhc/mzp002 19190135 10.1093/intqhc/mzp002

[38] Boller C, Wyss K, Mtasiwa D, Tanner M (2003) Quality and comparison of antenatal care in public and private providers in the United Republic of Tanzania. Bulletin of the World Health Organization 81: 116–122. 12751419 PMC2572401

[39] Hulton LA, Matthews Z, Stones RW (2007) Applying a framework for assessing the quality of maternal health services in urban India. Social Science & Medicine 64: 2083–2095. 17374551 10.1016/j.socscimed.2007.01.019

[40] Gross K, Alba S, Glass TR, Schellenberg JA, Obrist B (2012) Timing of antenatal care for adolescent and adult pregnant women in south-eastern Tanzania. BMC pregnancy and childbirth 12. 10.1186/1471-2393-12-16 PMC3384460 22436344

[41] Haddrill R, Jones GL, Mitchell CA, Anumba DO (2014) Understanding delayed access to antenatal care: a qualitative interview study. BMC Pregnancy and Childbirth 14. 10.1186/1471-2393-14-207 PMC4072485 24935100

[42] Kumar S, Dansereau E (2014) Supply-side barriers to maternity-care in India: a facility-based analysis. PloS one 9: e103927 doi: 10.1371/journal.pone.0103927 25093729 10.1371/journal.pone.0103927 PMC4122393

[43] United Nations (2015) The Millennium Development Goals Report 2015. New York: United Nations

[44] Cresswell JA, Yu G, Hatherall B, Morris J, Jamal F, Harden A, et al (2013) Predictors of the timing of initiation of antenatal care in an ethnically diverse urban cohort in the UK. BMC Pregnancy and Childbirth 13. 10.1186/1471-2393-13-103 PMC3652742 23642084

[45] Phillimore J (2016) Migrant maternity in an era of superdiversity: New migrants’ access to, and experience of, antenatal care in the West Midlands, UK. Social Science & Medicine 148: 152–159. 26705910 10.1016/j.socscimed.2015.11.030

[46] Rowe RE, Magee H, Quigley MA, Heron P, Askham J, Brocklehurst P (2008) Social and ethnic differences in attendance for antenatal care in England. Public Health 122: 1363–1372. doi: 10.1016/j.puhe.2008.05.011 18639909 10.1016/j.puhe.2008.05.011

