# Correction: Analysis of socioeconomic differences in the quality of antenatal services in low and middle-income countries (LMICs)

**DOI:** 10.1371/journal.pone.0197203

**Published:** 2018-05-07

**Authors:** Joshua Amo-Adjei, Kofi Aduo-Adjei, Christiana Opoku-Nyamaah, Chimaraoke Izugbara

The third and fourth authors’ names are spelled incorrectly. The correct names are: Christiana Opoku-Nyamaah and Chimaraoke Izugbara. The correct citation is: Amo-Adjei J, Aduo-Adjei K, Opoku-Nyamaah C, Izugbara C (2018) Analysis of socioeconomic differences in the quality of antenatal services in low and middle-income countries (LMICs). PLoS ONE 13(2): e0192513. https://doi.org/10.1371/journal.pone.0192513
